# 

**Published:** 1855-04

**Authors:** 


					﻿No. XI.]
APRIL.
[Vol. X.
BUFFALO
MEDICAL JOURNAL
AMD
Jltontl)li) Ucuictv
»
MEDICAL AND SURGICAL SCIENCE.
AUSTIN FLINT, M. D., i
SANFORD B. HUNT, M. D., J Editobs-
BUFFALO, N. Y.:
PUBLISHED BY THOMAS <k LATHBOP8.
Commercial Advertiser Buildings
1855.
Price 82 JO per annum, in advance.
				

